# Shaping Ability of the ProTaper Gold and R-Motion Systems in Long-Oval Canals: A Microcomputed Tomography Study

**DOI:** 10.1155/tswj/5825229

**Published:** 2025-03-06

**Authors:** Marina da Cunha Isaltino, Wesley Viana de Sousa, Christianne Tavares Velozo Telles, Luiza de Almeida Souto Montenegro, Hugo Victor Dantas, Frederico Barbosa de Sousa, Diana Santana de Albuquerque

**Affiliations:** ^1^Postgraduate Program in Dentistry, University of Pernambuco (UPE), Recife, Brazil; ^2^Postgraduate Program in Dentistry, University of Paraíba (UFPB), João Pessoa, Brazil

**Keywords:** long-oval canal, microcomputed tomography, root canal preparation, untouched area

## Abstract

**Objectives:**The aim of this study was to evaluate, by microcomputed tomography (micro-CT), the shaping ability and reduction of dentin thickness in mesial and distal surfaces of the ProTaper Gold (PTG; Dentsply Sirona, Ballaigues, Switzerland) and R-Motion (RM; FKG Dentaire, La Chaux-de-Fonds, Switzerland) systems in long-oval canals of lower incisors.

**Materials and Methods:** Twenty long-oval lower incisors were selected, compared anatomically, and scanned by micro-CT (SkyScan 1172, Bruker Micro-CT, Kontich, Belgium). The teeth were divided into two groups (*n* = 10) according to instrumentation technique: PTG and RM. The following morphometric measurements were made on images recorded before and after preparation: volume, surface area, structure model index (SMI), unprepared walls, and dentin thickness parameters were calculated. The Shapiro–Wilk test and Student *t*-test were used for comparison of the data between the two groups at a significance level of 5%.

**Results:** Significant differences were found in volume (127.60% vs. 69.22%) and reduction in dentin thickness on the distal surface of the root canal, with higher values for PTG compared to RM, respectively (*p* < 0.05). There was no significant difference in surface area (34.79% vs. 26.93%), SMI, and the percentage of unprepared areas (9.43% vs. 12.22%).

**Conclusion:** Although GTP resulted in a greater reduction in dentin thickness on the distal surface and volume, there was no difference in the percentage of contact surface and unprepared areas between the two systems, confirming that neither technique was able to completely prepare the long-oval canals of the lower incisors.

**Clinical Relevance:** Despite the technological advances in instruments launched on the market, exhibiting greater flexibility due to their metallurgical properties and short sequences, there is little scientific evidence regarding the new RM instrument, manufactured by FKG Dentaire, and its root canal modeling capacity.

## 1. Introduction

The development of automated instrumentation was a major advance in root canal preparation in recent years, providing more flexible instruments with better shaping ability of the root canal system (RCS). This advancement, in turn, led to a significant decrease in working time, which is an important benefit in endodontic practice [1, 2]. The main goals of chemical–mechanical preparation are to clean, shape, and disinfect the root canal [3]. Despite many technical advances in endodontics, the complex anatomical variation of root canals continues to pose a challenge during the endodontic preparation of the root canal, and its complex anatomical variation continues to pose a challenge, especially in the case of oval, flat, and curved canals [4]. The continuous rotary and reciprocating systems available have failed to improve the debridement of oval canals, resulting in large areas of unprepared walls and in the accumulation of debris in isthmuses and irregularities along the root canal space [5–7].

The variety of commercially available instruments is increasing, including innovations in the design of smaller and varied tapers with a finer wire diameter, in an attempt to preserve the natural position of the root canal and to promote more conservative dentin wear during preparation. Within this context, studies have shown that the preservation of the pericervical dentin significantly reduces traction forces and the risk of root fracture [1, 8–10]. In addition to heat treatment of nickel–titanium (NiTi) alloys and hybridization strategies to treat more complex cases [9, 11], some instruments have been modified to improve their physical shape and properties and to permit their use in both the reciprocating and the continuous rotary mode. However, more conclusive studies are needed to provide more accurate assessments of the shaping ability of these instruments [7].

Since the introduction of NiTi rotary instruments, an increasing number of rotary systems have been commercialized by various manufacturers. Among them are the new generations of the ProTaper Gold (PTG) and ProTaper Ultimate (Dentsply Maillefer, Ballaigues, Switzerland) systems [12]. The PTG has the same geometry as the ProTaper Universal (Dentsply Maillefer, Ballaigues, Switzerland), with a convex triangular cross-section and progressive conicity, increasing efficiency and safety in cutting. Its differential feature is the gold heat treatment, which adds greater flexibility to the instrument and reduces the risk of fracture [13].

In 2020, the R-Motion system [14] was launched, featuring reciprocal kinematics and a triangular cross-section made from NiTi Max Wire heat treatment, which allows for better centralization and respect for canal anatomy. The reduced core sizes of RM instruments are less invasive than equivalent competitive files, decreasing the risk of excessive dentin removal and minimizing the impact on uninfected areas [15, 16]. Its main characteristic is the shape profile, with lower conicity (0.04 and 0.06), which distinguishes it from alternative systems on the market and is tailored to the needs of each clinical case.

Along with advances in instruments and techniques, there has been considerable progress in the experimental methods used to analyze the results of root canal preparation in recent years [17]. Three-dimensional (3D) assessments are needed for a more accurate comparison of the shaping ability of instruments [7]. Microcomputed tomography (micro-CT) has been established as an accurate and nondestructive imaging method for evaluating the effects of biomechanical preparation on canal anatomy and for quantitative assessment and qualitative morphological analysis of root canals [18, 19].

Therefore, the aim of this study was to evaluate, by micro-CT, the shaping ability of the R-Motion (FKG Dentaire, La Chaux-de-Fonds, Switzerland) and PTG (Dentsply Maillefer, Ballaigues, Switzerland) systems in long-oval canals of lower incisors in terms of the following criteria: increase in volume and surface area, structure model index (SMI), percentage of unprepared areas, and percentage of removed dentin from the mesial and distal surfaces. The PTG system is well established in the endodontic literature, and its characteristics differ from those of the RM system. Few studies have so far analyzed the performance of these instruments, and the null hypothesis tested is that there would be no significant difference in the cited parameters between the two systems during root canal preparation.

## 2. Materials and Methods

### 2.1. Sample Size Calculation

Sample size calculation for the present study was conducted according to de Carvalho et al. [15], who used a similar methodology. The sample size was based on parameters determined with a margin of error of 5%, a power of 80%, and an effect size of 1.64. The calculations performed with SigmaPlot 11.0 (Systat Software Inc.) indicated a minimum number of eight specimens per group. Ten specimens were included in each group to compensate for possible loss during the study.

### 2.2. Selection and Characterization of the Sample

The ethics committee of the University of Pernambuco (UPE) approved the research project (Protocol: 5.929.259). Eighty extracted human single-rooted lower incisors were obtained from the Human Tooth Bank of the Tabosa de Almeida University Center (ASCES/UNITA), disinfected in 0.5% thymol solution (pH 7.0) at 4°C (Sigma-Aldrich, Darmstadt, Germany) for 7 days and stored in distilled water under refrigeration. After radiographic evaluation (Saevo Gnatus, Equipamentos Médico-Odontológico Ltda., Ribeirão Preto, SP, Brazil), taken from both buccolingual and mesiodistal directions of each specimen and application of pre-established inclusion and exclusion criteria, 30 single-rooted mandibular incisors Vertucci Type I were selected, and their crowns were sectioned without exposing the coronary pulp with diamond burs (n.3071, KG Sorensen Ind. e Com. Ltda., Cotia, SP, Brazil) coupled to a high-speed handpiece (Dabi-Atlante, Ribeirão Preto, SP, Brazil) and standardized to an average length of 16 mm. The specimens were then submitted to baseline micro-CT scanning with the SkyScan 1172 system (Bruker Micro-CT, Kontich, Belgium) at the Laboratory of Microscopy and Biological Imaging (LAMIB) of the Department of Morphology, Federal University of Paraíba (UFPB). The following parameters were used for image acquisition: 100 kV, 100 *μ*A, 180° rotation, and a 0.6° rotation step, resulting in images with a voxel size of 26.8 *μ*m. After the acquisition of the images, the NRecon program (Version 1.5.23, Bruker Micro-CT) was used to reconstruct the cross-sections using the following parameters: smoothing level of 5, ring artifact correction of 6, and beam hardening correction of 25%. Preoperative 3D models of the root canals were processed for the evaluation of canal anatomy. The homogeneity of the morphological data (volume and surface area) for the 30 canals was confirmed statistically (*p* > 0.05). The shape of the canal was classified by calculating the aspect ratio, defined as the ratio of the major (*D*) to the minor (*d*) diameter of all sections, measured at the same site 10 mm from the apex. A long-oval shape was defined when the *D*/*d* ratio was greater than 2, or two times higher than the measurement taken at right angles [20]. For baseline scanning, the teeth were inserted into Adsil addition silicone (Vigodent Coltene, Rio de Janeiro, Brazil), with the root facing upwards. This material was fixed to the rotating table with utility wax (New Wax, Technew, Rio de Janeiro, Brazil) and positioned parallel to the radiation source, thus reducing possible movement during image capture.

### 2.3. Biomechanical Preparation of Root Canals

To minimize bias, 20 single-rooted lower incisors classified as long oval were selected and divided into two groups of 10 teeth each based on morphological features of the RCSs. All biomechanical preparation procedures were performed by a single previously trained endodontic specialist with more than 15 years of experience in mechanical instrumentation using an X-Smart Plus motor (Dentsply Maillefer). However, since the two instruments are operated differently, the operator was not blinded to the instrumentation technique. To simulate the resistance imposed by the periodontal ligament and to prevent the formation of vapor lock in the canal, the apices were sealed with a gingival barrier gel (TOP Dan, FGM, Joinville, SC, Brazil) and then inserted into a dental manikin (FKG Dentaire, La Chaux de Fonds, Switzerland) to mimic clinical conditions. Crown access was achieved under an operating microscope at 16x magnification (Alliance, São Carlos, SP, Brazil), and the samples were numbered and divided into two experimental groups (*n* = 10) according to the type of chemical–mechanical preparation technique: PTG and R-Motion.

#### 2.3.1. PTG Group

The root canals were first irrigated with 2 mL of 2.5% NaOCl (Asfer, São Caetano do Sul, SP, Brazil) using a screw-on syringe (Ultradent, Joinville, SC, Brazil) and a NaviTip needle tip (Ultradent). Patency was achieved with a #10 C-Pilot file (VDW, Munich, Germany); when the type of the instrument was visible through the main foramen, 1.0 mm was subtracted to determine the working length (WL). The instruments were inserted into the canal with insertion/removal movements three times at a controlled amplitude of approximately 3 mm by applying light pressure against the walls. The S1 (18/0.02), S2 (20/0.04), F1 (20/0.07), F2 (25/0.08), and F3 (30/0.09) instruments were used sequentially (300 rpm), with variable torque according to manufacturer instructions (S1: 4.0, S2 and F1: 1.50, and F2 and F3: 3.0), under continuous rotary movement until the WL was reached. Patency was confirmed, and the root canal was washed with 2 mL of 2.5% NaOCl (1 min) after each instrument change. The smear layer was removed by irrigation with 5 mL of 17% ethylenediaminetetraacetic acid (EDTA) (Maquira, São Paulo, Brazil) (2 min), followed by final irrigation with 5 mL of 2.5% NaOCl (2 min) and drying with Cell Pack sterile-absorbent paper points (Tanari, Manaus, AM, Brazil) whose size was equivalent to the final instrument. The PTG file was discarded every two teeth.

#### 2.3.2. R-Motion Group

The root canals were first irrigated with 2 mL of 2.5% NaOCl (Asfer, São Caetano do Sul, SP, Brazil) using a screw-on syringe (Ultradent, Joinville, SC, Brazil) and a NaviTip needle tip (Ultradent). Patency was achieved with a #10 C-Pilot file (VDW, Munich, Germany); when the type of the instrument was visible through the main foramen, 1.0 mm was subtracted to determine the WL. The instruments were inserted into the canal with insertion/removal movements three times at a controlled amplitude of approximately 3 mm by applying light pressure against the walls. Next, the glider instrument (15/0.03) was applied in reciprocating movement using the prefabricated RECIPROC ALL configuration until the WL was reached. Patency was confirmed, and the root canal was washed with 2 mL of 2.5% NaOCl (1 min) after each instrument change. The R-Motion instrument (30/0.04) was used to instrument root canals until the WL was reached. After patency was achieved, the root canal was washed with 2 mL of 2.5% NaOCl (1 min). The smear layer was removed by irrigation with 5 mL of 17% EDTA (2 min) (Maquira, São Paulo, Brazil), followed by final irrigation with 5 mL of 2.5% NaOCl (2 min) and drying with Cell Pack sterile-absorbent paper points (Tanari, Manaus, AM, Brazil), whose size was equivalent to the final instrument. The R-Motion file was discarded every two teeth.

### 2.4. Final Micro-CT Scanning

After root canal preparation, the teeth were scanned again on the micro-CT device using the same parameters as described above. After reconstruction of the microtomographic images from the scans, the DataViewer program (Bruker Micro-CT) was used for registering the sets of images in order to align them geometrically in the same spatial position using the 3D registration function. Thus, a new registration of pre- (reference) and postinstrumentation (target) image data, now saved in new folders, was obtained for both time points. The images were reconstructed using the NRecon program (SkyScan, Kontich, Belgium). The image file was then opened in the CTAn v.1.16.1 program (Bruker Micro-CT), and the TOP (apical foramen where the canal could still be identified) and BOTTOM (just below the cementoenamel junction (CEJ)) were determined. Using the predetermined TOP and BOTTOM, the region of interest (ROI) of the image in the present study was selected. As a default, TOP was defined as the first selected slice, chosen when it was 1 mm short of the apical end; the last selected slice, BOTTOM, was chosen considering the CEJ. Next, a geometric figure was selected to enable the interpolation of the ROIs to make them united into a single structure.

The next step was the binarization of the initial image, that is, the transformation into a black-and-white image by adjusting the histogram values so that the binarized image was similar to the original in terms of canal volume. The increase in canal volume and surface area and the SMI were then analyzed. The SMI characterizes the geometry of the root canal as having a rod-like or plate-like shape on a scale from 0 to 3. The lower the value, the flatter the canal; the closer the value is to 3, the more rounded the canal [18]. Using the custom processing option, a task list was created to execute mathematical functions for personalized processing of the images of untouched surfaces, which provided the baseline surface (BS) area of the root canal wall in square millimeters and the untreated surface (US) area. The percentage of the US area in relation to the BS area was calculated according to the formula: %*US* = (*US*∗100/*BS*). The percentage volume of dentin removal was calculated within the volume of interest (coronal) as follows: (*DVc*)/(*DVc* × 100), where *DVc* is the volume of dentin (in cubic millimeters) before and after preparation. Three trained observers performed the two assessments. The images were evaluated twice at an interval of 2 weeks; in the case of divergence, the images were analyzed together until a consensus was reached [21].

### 2.5. Statistical Analysis

The mean, standard deviation, median, and percent increase were calculated. Normality and equality of variances were assessed by the Shapiro–Wilk test and Levene's *F* test, respectively. Significant differences between the PTG and RM groups in terms of volume, surface area, SMI, unprepared areas, and proximal wall diameter after root canal preparation were evaluated by the Student *t*-test with equal variances, paired Student's *t*-test, and paired Wilcoxon's test. The paired Student *t*-test was chosen for normally distributed data and the Wilcoxon test for variables that did not show a normal distribution. Statistical analysis was performed using SPSS 25 (IMB SPSS Inc., Chicago, IL, United States), adopting a level of significance of 5%.

## 3. Results

**Figure 1:**
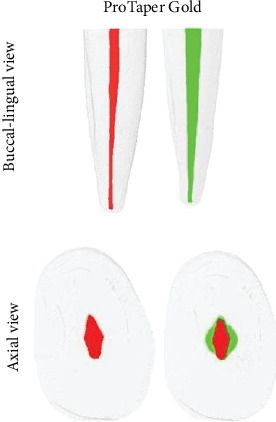
Buccal-lingual and axial view of three-dimensional reconstructions of lower incisors. Red corresponds to images before, and green corresponds to images after preparation with the ProTaper Gold.

**Figure 2:**
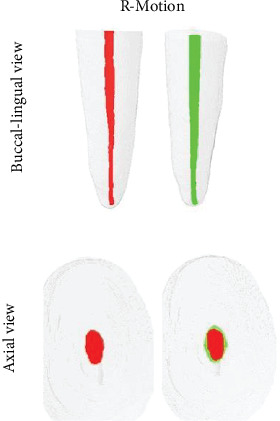
Buccal-lingual and axial view of three-dimensional reconstructions of lower incisors. Red corresponds to images before, and green corresponds to images after preparation with the R-Motion.

The results are reported as the mean and standard deviation (mean ± SD) and median and 25th and 75th percentiles (median [P25; P75]), depending on the distribution of the data.  Table 1 shows the results of the 3D parameters (changes in volume, surface area, and SMI) and the percentage of unprepared areas. The dentin thickness analysis of the mesial and distal of the 20 lower incisors before and after preparation with the different systems (PTG and R-Motion) is shown in Table 2. As can be seen in Table 1, the baseline morphological features of the root canals were homogenous (*p* > 0.05). Significant differences in volume (126.60% vs. 69.22%) and a greater reduction in dentin thickness on the distal surface of the root canal (15.96% vs. 0.11.25%) were observed after preparation, with higher values for PTG compared to RM, respectively (*p* < 0.05) (Tables 1 and 2). There was no statistically significant difference in surface areas (26.45% vs. 20.75%), SMI, or the percentage of unprepared areas (9.43% vs. 12.22%) (*p* > 0.05) after root canal preparation (Table 1). Figures 1 and 2 show representative images of the root canal before (red) and after (green) preparation with the two systems.

## 4. Discussion

The morphology of the RCS influences the instruments or techniques used to achieve successful endodontic treatment. Considering the effort necessary for the preparation of root canals with complex anatomy, the aim of this study was to compare the shaping ability of PTG, a well-documented system, and of the new R-Motion system in long-oval canals of lower incisors, which pose a challenge in clinical practice. The null hypothesis is that there would be no difference in the performance of the two systems tested, which is confirmed in terms of surface area, the SMI parameter, and unprepared areas. However, a significant difference was found in volume and the reduction of dentin thickness in the distal wall of the channels.

The choice of anatomically compatible specimens is of fundamental importance when micro-CT is used for the comparison of results [22], enabling reliable two-dimensional and 3D nondestructive longitudinal analysis based on more accurate measurements [23]. Micro-CT is the gold standard for the analysis of anatomical morphology [24]. The voxel size determines the level of detail that an image provides, that is, its spatial resolution. If the spatial resolution of an image is increased by decreasing the voxel size, a higher dose of radiation is required to achieve the same noise level as the larger voxel size [8]. To ensure anatomical standardization of the specimens, the baseline root canal volume was calculated and statistically evaluated in previous studies using micro-CT [24]. Furthermore, these studies used the classification of long-oval canals for sample selection because of their anatomical complexity. Lower incisors were used in the present study. Jou et al. [20] proposed a method for the definition of long-oval-shaped canals that consists of buccal-lingual and mesiodistal measurements of the canal. A long-oval shape is defined when the canal dimension exceeds two to four times the smallest measurement.

Considering the importance of defining the characteristics of the sample and of correct evaluation, the SMI is a recommended tool for characterizing the geometry of trabecular structures as plate-like or rod-like [21]. This index is commonly used to analyze the geometry of root canals and to measure surface convexity as the volume increases [6]. In the present study, both systems promoted an increase in SMI values after the preparation of long-oval canals, providing a more conical shape of the canal, with no significant difference in the mean percent increase in these values. Interestingly, despite the smaller taper of the R-Motion instrument, its larger conical active tip design increases cutting efficiency and penetration while, at the same time, providing space for the instrument in order to maintain the original anatomical path of the canal [15].

Bacterial biofilms and tissue remnants can persist in unprepared areas [25] and consequently compromise treatment outcomes [18]. In the present study, neither system was able to fully prepare the root canal, in agreement with previous studies [26–30]. Although following a trend towards minimally invasive preparation and smaller taper, the R-Motion reciprocating instrument [15] did not promote a significant difference in the percentage of unprepared areas compared to the PTG rotary instrument, with percentages of 12.22% and 9.43%, respectively.

Analysis of root canal area parameters after preparation with the two systems showed a volume increase compared to the original anatomy, with respective percentages of 127.60% and 69.22% for PTG and R-Motion. Previous studies evaluating different mechanical systems also indicate a significant increase in canal surface PTG [30–32]. However, the percentages ranged from 1.24% to 33% and cannot be compared with the present study because of methodological differences, including the type of tooth, canal shape, and instrument investigated. The comparison between the systems regarding the increase in volume and final surface area of the root canal revealed a significant difference, with the PTG group showing higher percentage values than the RM group. The analysis of 3D parameters demonstrated a greater increase in surface area and volume for the PTG instrument, due to its higher conicity, which allowed for better modeling of the canals using the rotary instrument sequence (Sx–F3), ending with a 30/0.09 instrument. This instrument has an active part with varying conicity, starting at a 3 mm diameter (D0–D3) and a final diameter of 1.20 mm at D16 [33], which justifies the greater dentin wear and, consequently, the better modeling of the root canal. In contrast, the RM reciprocating group was initially prepared with the RM Glider and finished with a 30/0.04 instrument, which has fixed conicity (D0–D16) and a final diameter of 0.94 mm [15]. Although the surface area and volume move in the same direction, the proportion may be different, depending on the standard anatomical conicity of the root canal, as in the present study, where the RM instrument presented a significant contact surface area, as it is well adjusted to the same, but does not create volume, compared to the varied taper instrument.

Preparation of the RCS is currently being guided by the concept of minimally invasive endodontics, which consists of the development of instruments with smaller diameters and tapers in an attempt to preserve the dentin in the cervical and apical regions of the teeth [34]. Pericervical dentin is defined as the area located approximately 4 mm above the bone crest, which is responsible for the transmission of occlusal forces to the root [35]. Within this context, the present study compared the reduction in proximal (mesial and distal) wall diameter after the preparation of long-oval canals with R-Motion instruments, whose characteristics meet this new trend, and PTG instruments, which are supported by scientific evidence. Significant differences were found between the systems tested, with a greater reduction in dentin thickness in the distal margin of the root canal. However, there was a safety margin that prevented the compromise of these thin walls that the anatomy presents.

Shaping ability plays a fundamental role in the capacity of the irrigating solution to exert its desired effect on all thirds of the canal and consequently promote better decontamination [36]. In addition to advances in the root canal preparation technique, decontamination approaches have been the focus of studies investigating the activation of irrigating solutions with ultrasonic inserts in order to increase penetration in difficult-to-access areas such as isthmuses, recesses, lateral canals, and apical deltas, which can compromise treatment [37].

Regarding limitations, studies evaluating shaping and disinfection after root canal preparation with different instruments are needed. For example, Loyola-Fonseca et al. [38] conducted an ex vivo study of the mesial roots of lower molars contaminated with a mixed bacterial culture to evaluate the antimicrobial effect and shaping ability of two instruments, complemented by ultrasonic activation of NaOCl, which is a notable alternative to evaluate the preparation area and to reduce microbial load.

Standardizing root canal morphology in micro-CT studies improves feasibility and minimizes possible biases interfering with the results [6]. The baseline morphological features of area and volume were homogenous in the present study. The PTG and R-Motion systems exhibited efficient shaping ability, with a significant difference being only observed in the percent increase in volume and surface area, as well as in the reduction in dentin thickness in the distal wall after preparation with PTG, due to its greater taper. This reinforces the extreme importance of a careful assessment of the compatibility of the instrument with the anatomy of the root canal during biomechanical preparation.

## 5. Conclusion

Based on the methodology used, the PTG and R-Motion systems demonstrated a difference in volume percentage and a greater reduction in dentin thickness in the distal surface walls of long-oval canals. However, there was no difference in the contact surface, unprepared areas, and SMI parameter, confirming that none of the techniques was able to fully prepare the long-oval canals of lower incisors.

## Figures and Tables

**Table 1:** Statistical comparison of volume, surface area, SMI, and percentage of unprepared areas before and after preparation and between the two groups.

**Variable**		**ProTaper Gold**	**R-Motion FKG**	**p** ** value**
		**M** **e** **a** **n** ± **SD**	**M** **e** **a** **n** ± **SD**	
		**Median (P25; P75)**	**Median (P25; P75)**	
Volume	Before	2.59 ± 0.85	2.30 ± 1.12	*p* = 0.513^a^
		2.51 (1.81; 3.41)	2.12 (1.32; 2.90)	
	After	5.50 ± 0.88	3.57 ± 1.41	*p* = 0.002^a^∗
		5.33 (4.73; 6.39)	3.22 (2.60; 4.51)	
	% increase	127.60 ± 66.26	69.22 ± 65.64	*p* = 0.005^b^⁣^∗^
		99.05 (80.83; 166.62)	44.36 (38.62; 64.91)	
	*p* value	*p* = 0.002^c^⁣^∗^	*p* = 0.001^c^⁣^∗^	

Surface area	Before	27.96 ± 6.02	22.70 ± 8.21	*p* = 0.119^a^
		28.05 (23.26; 30.46)	20.75 (14.85; 30.46)	
	After	34.79 ± 4.94	26.93 ± 8.77	*p* = 0.024^a^⁣^∗^
		34.42 (30.64; 37.03)	25.19 (19.71; 34.61)	
	% increase	26.45 ± 13.25	20.75 ± 16.33	*p* = 0.165^b^
		23.39 (16.65; 35.02)	16.61 (13.04; 20.38)	
	*p* value	*p* = 0.001^c^⁣^∗^	*p* = 0.002^c^⁣^∗^	

SMI	Before	1.76 ± 0.51	1.72 ± 0.57	*p* = 0.184^a^
		1.55 (1.33; 2.30)	2.34 (1.84; 2.79)	
	After	2.36 ± 0.36	2.11 ± 0.41	*p* = 0.169^a^
		2.29 (2.06; 2.69)	2.71 (2.11; 2.88)	
	% increase	38.27 ± 18.14	30.69 ± 41.62	*p* = 0.128^b^
		42.39 (17.14; 53.70)	17.11 (13.04; 29.55)	
	*p* value	*p* = 0.001^c^⁣^∗^	*p* = 0.005^c^⁣^∗^	

% unprepared areas (after)		9.43 ± 1.92	12.22 ± 3.91	*p* = 0.064^a^
		8.96 (7.88; 11.59)	12.03 (8.65; 15.48)	

Abbreviation: SMI = structure model index.

^a^Student's *t*-test with equal variances.

^b^Paired Wilcoxon's test.

^c^Paired Student's *t*-test.

⁣^∗^Significant difference at the 5% level.

**Table 2:** Statistical dentin thickness analysis of the mesial and distal surfaces before and after preparation between the two groups.

**Variable**		**ProTaper Gold**	**R-Motion FKG**	**p** ** value**
		**M** **e** **a** **n** ± **SD**	**M** **e** **a** **n** ± **SD**	
		**Median (P25; P75)**	**Median (P25; P75)**	
Mesial diameter	Before	1.46 ± 0.12	1.49 ± 0.20	*p* = 0.683^a^
		1.46 (1.36; 1.56)	1.50 (1.37; 1.52)	
	After	1.25 ± 0.13	1.31 ± 0.14	*p* = 0.372^b^
		1.27 (1.13; 1.34)	1.31 (1.18; 1.42)	
	% reduction	17.01 ± 7.77	14.21 ± 11.39	*p* = 0.529^a^
		15.44 (11.18; 23.62)	13.73 (6.19; 17.41)	
	*p* value	*p* = 0.001^c^	*p* = 0.004^c^⁣^∗^	

Distal diameter	Before	1.47 ± 0.16	1.45 ± 0.14	*p* = 1.000^a^
		1.42 (1.37; 1.54)	1.44 (1.37; 1.52)	
	After	1.27 ± 0.11	1.30 ± 0.14	*p* = 0.579^a^
		1.26 (1.19; 1.35)	1.29 (1.22; 1.36)	
	% reduction	15.96 ± 7.72	11.25 ± 4.91	*p* = 0.0121^a^
		14.96 (12.20; 22.80)	10.77 (7.63; 15.32)	
	*p* value	*p* = 0.001^c^⁣^∗^	*p* = 0.001^c^⁣^∗^	

^a^Student's *t*-test with equal variances.

^b^Paired Wilcoxon's test.

^c^Paired Student's *t*-test.

⁣^∗^Significant difference at the 5% level.

## Data Availability

The entire dataset that supports the results of this study has been published in the article itself.

## References

[1] Ahn S. Y., Kim H. C., Kim E. (2016). Kinematic effects of nickel-titanium instruments with reciprocating or continuous rotation motion: a systematic review of in vitro studies. *Journal of Endodontics*.

[2] Gomes M. S., Vieira R. M., Böttcher D. E., Plotino G., Celeste R. K., Rossi-Fedele G. (2021). Clinical fracture incidence of rotary and reciprocating NiTi files: a systematic review and meta-regression. *Australian Endodontic Journal*.

[3] Siqueira Junior J. F., Rôças I. N., Marceliano-Alves M. F., Pérez A. R., Ricucci D. (2018). Unprepared root canal surface areas: causes, clinical implications, and therapeutic strategies. *Brazilian Oral Research*.

[4] Versiani M. A., Leoni G. B., Steier L. (2013). Micro–computed tomography study of oval-shaped canals prepared with the self-adjusting file, Reciproc, WaveOne, and ProTaper Universal systems. *Journal of Endodontics*.

[5] Silva E. J. N. L., Belladonna F. G., Zuolo A. S. (2018). Effectiveness of XP-endo Finisher and XP-endo Finisher R in removing root filling remnants: a micro-CT study. *International Endodontic Journal*.

[6] Zuolo M. L., Zaia A. A., Belladonna F. G. (2018). Micro-CT assessment of the shaping ability of four root canal instrumentation systems in oval-shaped canals. *International Endodontic Journal*.

[7] Guedes I. G., Rodrigues R. C. V., Marceliano-Alves M. F., Alves F. R. F., Rôças I. N., Siqueira J. F. (2022). Shaping ability of new reciprocating or rotary instruments with two cross-sectional designs: an ex vivo study. *International Endodontic Journal*.

[8] Clark D., Khademi J. (2010). Modern molar endodontic access and directed dentin conservation. *Dental Clinics*.

[9] Gavini G., Santos M. ., Caldeira C. L. (2018). Nickel–titanium instruments in endodontics: a concise review of the state of the art. *Brazilian Oral Research*.

[10] Jiang Q., Huang Y., Tu X., Li Z., He Y., Yang X. (2018). Biomechanical properties of first maxillary molars with different endodontic cavities: a finite element analysis. *Journal of Endodontics*.

[11] De-Deus G., Belladonna F. G., Silva E. J. N. L. (2015). Micro-CT evaluation of non-instrumented canal areas with different enlargements performed by NiTi systems. *Brazilian Dental Journal*.

[12] Martins J. N. R., Silva E. J. N. L., Marques D. (2023). Characterization of the file-specific heat-treated ProTaper Ultimate rotary system. *International Endodontic Journal*.

[13] Elnaghy A. M., Elsaka S. E. (2016). Mechanical properties of ProTaper Gold nickel-titanium rotary instruments. *International Endodontic Journal*.

[14] FKG Dentaire, SA (2020). R-Motion-reciprocation the safest simply-Le Chaux – de Founs. https://www.fkg.ch/products/endodontics/canal-shaping-and-cleaning/r-motion.

[15] de Carvalho K. K. T., Petean I. B. F., Silva-Sousa A. C. (2022). Impact of several NiTi-thermally treated instrumentation systems on biomechanical preparation of curved root canals in extracted mandibular molars. *International Endodontic Journal*.

[16] Basturk F. B., Özyürek T., Uslu G., Gündoğar M. (2022). Mechanical properties of the new generation RACE EVO and R-Motion nickel–titanium instruments. *Materials*.

[17] de-Deus G., Rodrigues E. A., Belladonna F. G. (2019). Anatomical danger zone reconsidered: a micro-CT study on dentine thickness in mandibular molars. *International Endodontic Journal*.

[18] Perez A. R., Ricucci D., Vieira G. C. (2020). Cleaning, shaping, and disinfecting abilities of 2 instrument systems as evaluated by a correlative micro–computed tomographic and histobacteriologic approach. *Journal of Endodontics*.

[19] Sousa-Neto M. D., Silva-Sousa Y. C., Mazzi-Chaves J. F. (2018). Root canal preparation using micro-computed tomography analysis: a literature review. *Brazilian Oral Research*.

[20] Jou Y. T., Karabucak B., Levin J., Liu D. (2004). Endodontic working width: current concepts and techniques. *Dental Clinics*.

[21] Hildebrand T. O. R., Rüegsegger P. (1997). Quantification of bone microarchitecture with the structure model index. *Computer Methods in Biomechanics and Bio Medical Engineering*.

[22] Zuolo M. L., de-Deus G., Belladonna F. G. (2017). Micro–computed tomography assessment of dentinal micro-cracks after root canal preparation with TruShape and self-adjusting file systems. *Journal of Endodontics*.

[23] Gergi R., Osta N., Bourbouze G., Zgheib C., Arbab-Chirani R., Naaman A. (2015). Effects of three nickel titanium instrument systems on root canal geometry assessed by micro-computed tomography. *International Endodontic Journal*.

[24] Versiani M. A., Carvalho K. K. T., Mazzi-Chaves J. F., Sousa-Neto M. D. (2018). Micro–computed tomographic evaluation of the shaping ability of XP-endo Shaper, iRaCe, and EdgeFile systems in long oval-shaped canals. *Journal of Endodontics*.

[25] Siqueira J. F., Alves F. R. F., Versiani M. A. (2013). Correlative bacteriologic and micro–computed tomographic analysis of mandibular molar mesial canals prepared by self-adjusting file, Reciproc, and twisted file systems. *Journal of Endodontics*.

[26] Paqué F., Peters O. A. (2011). Micro–computed tomography evaluation of the preparation of long oval root canals in mandibular molars with the self-adjusting file. *Journal of Endodontics*.

[27] Guimaraes L. S., Gomes C. C., Marceliano-Alves M. F., Cunha R. S., Provenzano J. C., Siqueira J. F. (2017). Preparation of oval-shaped canals with TRUShape and Reciproc systems: a micro–computed tomography study using contralateral premolars. *Journal of Endodontics*.

[28] Velozo C., Silva S., Almeida A. (2020). Shaping ability of XP-endo Shaper and ProTaper Next in long oval-shaped canals: a micro-computed tomography study. *International Endodontic Journal*.

[29] Alves F. R. F., Andrade-Junior C. V., Marceliano-Alves M. F. (2016). Adjunctive steps for disinfection of the mandibular molar root canal system: a correlative bacteriologic, micro—computed tomography, and cryopulverization approach. *Journal of Endodontics*.

[30] Silva E. J., Lima C. O. ., Barbosa A. F. A., Lopes R. T., Sassone L. M., Versiani M. A. (2022). The impact of TruNatomy and ProTaper Gold instruments on the preservation of the periradicular dentin and on the enlargement of the apical canal of mandibular molars. *Journal of Endodontics*.

[31] de Medeiros T. C., de Lima C., Barbosa A. F. (2021). Shaping ability of reciprocating and rotary systems in oval-shaped root canals: a microcomputed tomography study. *Acta Odontológica Latinoamericana*.

[32] Versiani M. A., Pécora J. D., Sousa-Neto M. D. (2013). Microcomputed tomography analysis of the root canal morphology of single-rooted mandibular canines. *International Endodontic Journal*.

[33] Markvart M., Darvann T. A., Larsen P., Dalstra M., Kreiborg S., Bjørndal L. (2012). Micro-CT analyses of apical enlargement and molar root canal complexity. *International Endodontic Journal*.

[34] Aazzouzi-Raiss K., Ramírez-Muñoz A., Mendez S P. M., Vieira G. C. S., Aranguren J., Pérez A. R. (2023). Effects of conservative access and apical enlargement on shaping and dentin preservation with traditional and modern instruments: a micro-computed tomographic study. *Journal of Endodontics*.

[35] Clark D., Khademi J., Herbranson E. (2013). Fracture resistant endodontic and restorative preparations. *Dentistry Today*.

[36] Usman N., Baumgartner J., Marshall J. (2004). Influence of instrument size on root canal debridement. *Journal of Endodontics*.

[37] Lee O. Y. S., Khan K., Li K. Y. (2019). Influence of apical preparation size and irrigation technique on root canal debridement: a histological analysis of round and oval root canals. *International Endodontic Journal*.

[38] Loyola-Fonseca S. C., Campello A. F., Rodrigues R. C. V. (2023). Disinfection and shaping of Vertucci class II root canals after preparation with two instrument systems and supplementary ultrasonic activation of sodium hypochlorite. *Journal of Endodontics*.

